# The relationship between coping strategies with sexual satisfaction and sexual intimacy in women with multiple sclerosis

**DOI:** 10.1186/s12991-023-00451-4

**Published:** 2023-05-25

**Authors:** Samaneh Imanpour Barough, Hedyeh Riazi, Zohreh Keshavarz, Maliheh Nasiri, Ali Montazeri

**Affiliations:** 1 Students Research Office, School of Nursing and Midwifery, Shahid Beheshti University of Medical Sciences, Tehran, Iran; 2 Department of Midwifery and Reproductive Health, School of Nursing and Midwifery, Shahid Beheshti University of Medical Sciences, Tehran, Iran; 3 Department of Biostatistics, School of Paramedical Sciences, Shahid Beheshti University of Medical Sciences, Tehran, Iran; 4 Population Health Research Group, Health Metrics Research Center, Iranian Institute for Health Sciences Research, ACECR, Tehran, Iran; 5 Faculty of Humanity Sciences, University of Science and Culture, Tehran, Iran

**Keywords:** Coping strategies, Sexual satisfaction, Sexual intimacy, Multiple Sclerosis, Women

## Abstract

**Background:**

Multiple sclerosis profoundly affects the sexual aspects of patients’ life, especially in women. Various coping strategies are used by women with multiple sclerosis to overcome, tolerate, or minimize these sexual effects. The present study aimed to assess the relationship between sexual satisfaction, sexual intimacy, and coping strategies in women with multiple sclerosis.

**Methods:**

This cross-sectional study was performed on a sample of 122 married women who were members of Iran’s MS society in Tehran, Iran. The study was conducted from December 2018 to September 2019. Data were collected using the Index of Sexual Satisfaction (ISS), the Sexual Intimacy Questionnaire (SIQ), and the Folkman and Lazarus Coping Strategies Questionnaire. Frequency, percentage, mean and standard deviation were used to explore the observations. Independent t-test and logistic regression were applied to analyze the data using the SPSS-23.

**Results:**

The majority (*n* = 71, 58.2%) used an emotion-focused coping strategy with the highest score for the escape-avoidance subscale [mean (SD): 13.29 (5.40)]. However, 41.8% of the patients (*n* = 51) used a problem-focused coping strategy with the highest score for the positive reappraisal strategy subscale [mean (SD): 10.50 (4.96)]. The sexual satisfaction in women with problem-focused coping strategies was significantly higher than women who used emotion-focused coping strategies (95.6 vs. 84.71, *P*-value = 0.001). There was a negative association between sexual intimacy and higher emotion-focused coping strategy (OR = 0.919, 95% CI 0.872–0.968, *P* = 0.001).

**Conclusions:**

Problem-focused coping strategy in women with multiple sclerosis increases sexual satisfaction, while the emotion-focused coping strategy has a significant negative relationship with sexual intimacy.

## Introduction

Multiple sclerosis (MS) is a central nervous system disease that is common in women of reproductive age. The disease leads to progressive demyelination and destruction of nerves and neurons [1] and significantly affects physical, sexual, and mental health [2]. The incidence rate of MS is increasing. It is estimated that 2.8 million people are living with MS worldwide [3], and its latest prevalence in Iran and Tehran (the capital) is 148.06 and 162.38 per 100,000, respectively [4, 5]. The prevalence of MS in women is three times higher than in men [6].

Sexual disorders have been reported in 34–85% of women with MS [6, 7]. Neurological and psychological factors, depression, side effects of medications, and physical problems such as fatigue, muscle weakness, changes in menstruation, pain, and concerns about urinary and bowel incontinence are some of the factors affecting sexual disorders [8]. Sexual disorders affect patients’ sexual health [9]. Since sexual health plays an important role in improving women’s quality of life, it is necessary to pay particular attention to this issue [1].

Sexual satisfaction and sexual intimacy are considered the main components of sexual health [10]. Sexual satisfaction is a physical and emotional response to a person’s assessment of sex that emphasizes the positive or negative feelings after intercourse and the fulfillment of sexual needs of the person and his/her partner [11]. Numerous studies have reported the effect of sexual satisfaction on marital satisfaction, quality of life, and women’s self-esteem [12, 13]. Studies have shown that MS reduces sexual satisfaction in women through physical changes, sexual disorders (decreased genital nerve conduction, orgasm disorders), and psychological effects (fatigue, depression, and anxiety) [10, 14]. This can cause many unfortunate consequences such as coldness between couples, feelings of inferiority, lack of self-confidence, and depression in women with MS [7].

Sexual intimacy is another factor that can be affected by MS [1]. It refers to sharing romantic experiences, the need for body contact and sexual intercourse which is planned for sexual arousal and satisfaction. Studies have shown that neurological disorders such as MS can directly or indirectly affect women’s the sexual health and sexual function [8]. It is argued that following physical and psychological disorders caused by MS, sexual dysfunction [8] decreased sexual arousal, and decreased sexual desire [14] is inevitable. In turn, these could lead to a decrease in sexual intimacy in women, which is one of the most important concerns in women with MS [6].

There are various coping strategies in different age groups, at different stages of life, or among different patient populations. In general, coping strategies include the mental, emotional and behavioral efforts of individuals that are used to overcome or minimize the effects of stress when faced with mental pressure. The purpose of these strategies is to manage and change the source of stress (problem-focused strategies) or to regulate stressful emotions (emotion-focused strategies) [15].

Women with MS usually use coping strategies such as problem-solving, emotional release, and avoidance strategies; to adapt and meet their own impaired needs [16]. Several studies investigated the relationship between using different coping strategies by multiple sclerosis patients and a variety of issues such as mood profile [17], psychological outcomes [18], employment [19], and burden of disease [20].

It is believed that women with MS who benefit from medication, psychological counseling, and using active and positive coping strategies have a faster treatment process and achieve the desired state of physical, psychological, and social health in a short time [16, 21]. Conversely, a study on coping strategies used by patients with relapsing multiple sclerosis found that maladaptive coping strategies used by MS patients were associated with relevant clinical aspects of the disease and with worse health-related quality of life [22]. As pointed out by some investigators, using less avoidant and cognitively demanding adaptive coping strategies may improve the quality of life in MS patients [23]. Also, a recent study indicated that the use of coping strategies in multiple sclerosis is associated with demographic and clinical characteristics [24]. However, although various factors of quality of marital life and sexual disorders caused by MS have been investigated in these patient populations [25–27], to the best of our knowledge, the relationship between coping strategies and important factors related to the quality of marital life, including sexual satisfaction and sexual intimacy has not been studied yet. Thus, considering the importance of identifying coping strategies used by MS patients, the present study was conducted to determine the relationship between sexual satisfaction and sexual intimacy with the type of coping strategies in women with MS. More specifically, we were interested to know which type of coping strategy could be helpful to improve sexual satisfaction and sexual intimacy in women who are suffering from MS to be able to provide evidence for appropriate interventions.

## Methods

### Study design

The present cross-sectional study was performed on a sample of women with MS in Tehran, Iran. A convenience sampling method was used. The study was conducted from December 2018 to September 2019.

### Participants and setting

Participants were married women who suffered from MS and were members of Iran’s MS society. To recruit patients, we contacted the society and introduced the project. They provided a list of married female members living in Tehran, Iran. The first investigator contacted women and explained the study and those who fulfilled the inclusion criteria and were interested in participating were entered into the study. The continuous sampling method was used to obtain the calculated sample size. The inclusion criteria were being literate, married for at least six months, having sexual activity, and no physical disability. The exclusion criteria were partial completion of questionnaires and having a spouse with sexual dysfunction (according to the woman’s statement).

### Sample size calculation

Considering the least correlation between coping strategies with sexual satisfaction and sexual intimacy (r = 0.3) the following formula was used to calculate sample size:

$$n \ge \left[ {\frac{{\left( {z_{1 - \alpha /2} + z_{1 - \beta } } \right)}}{{0.5 \times \ln \left[ {\left( {1 + r} \right)/\left( {1 - r} \right)} \right]}}} \right]^{2} + 3$$

where *α* = 0.05, *β* = 0.10 (power 90%) a sample size of 112 women was achieved. However with a 10% drop out a sample of 122 women was thought [28].

### Data collection

The following questionnaires were used to collect the data:

1. Index of sexual satisfaction (ISS): this was developed by Hudson et al. in 1981 [29]. The ISS consists of 25 questions, and the response categories are rated on a 5-point Likert scale ranging from 1 to 5 (never, rarely, sometimes, most of the time, and always). The questionnaire includes both positive aspects (12 items such as ‘I feel that my partner enjoys our sex life’) and negative aspects (13 items such as ‘My partner does not satisfy me sexually’). The score for the questionnaire ranges from 125 (maximum) to 25 (minimum). The score on the questionnaire could be categorized as follows: a score below 60 as poor sexual satisfaction, a score between 60 and 90 as moderate sexual satisfaction, and a score above 90 as high sexual satisfaction. The validity of this questionnaire has been confirmed in various studies [10, 22, 30]. We used a validated Persian version of the questionnaire [30]. The reliability of the questionnaire for the current study was 0.89, as estimated by Cronbach’s alpha coefficient.
2. The Sexual Intimacy Questionnaire (SIQ): it was developed by Shahsiah et al. in Iran (2010) based on the Bagarozi sexual intimacy questionnaire [31] and consists of 30 items such as ‘We talk to each other about the quality of our sexual relationship’ and ‘I could not express my sexual feeling to my partner’. Each item is rated on a 4-point Likert scale from ‘always’ to ‘never’ giving maximum and minimum scores of 120 and 30, respectively. A score less than 60 indicates poor, between 60 and 90 moderate, and above 90 as high sexual intimacies [11]. The validity of the questionnaire has been confirmed by various studies [11]. The internal consistency of the questionnaire for the current study was 0.90 as calculated by the Cronbach’s alpha coefficient.
3. Folkman and Lazarus Coping Strategies Questionnaire: this questionnaire was developed by Folkman and Lazarus in 1986 [15] and consists of eight subscales tapping into two coping strategies: problem-focused strategies (seeking social support, accepting responsibility, planful problem-solving, and positive reappraisal) and emotion-focused strategies (confrontive coping, distancing, escape-avoidance, self-controlling). The questionnaire contains 66 items where each item is rated on a Likert scale ranging from zero ‘I have not used at all’ to 3 ‘I have used a lot’. We used a validated Persian version of the questionnaire [32]. As estimated by Cronbach's alpha coefficient, the internal consistency of the questionnaire in the current study was 0.92 for problem-focused coping strategies and 0.88 for emotion-focused coping strategies.

### Data analysis

The data obtained from the questionnaires were analyzed using SPSS version 23. Statistical data were described by calculating indices of central tendency and dispersion. To assess the relationship between coping strategies and outcome variables (that are, sexual satisfaction and sexual intimacy), binary logistic regression analyses were performed. As such proportional to the mean score of sexual satisfaction and sexual intimacy, respondents were categorized into two groups: those who scored equal or higher than mean and those who scored less than mean. Age, education, duration of marriage, number of children, frequency of sexual intercourse per week before and after the disease, and coping strategies were entered into the logistic model as independent variables. Odds ratio (and 95% confidence intervals) for likelihood of higher sexual satisfaction and sexual intimacy were reported. The significant level was set at *P* < 0.05.

### Ethics approval and consent to participate

The ethics committee of Shahid Beheshti University of Medical Sciences approved the study (IR.SBMU.PHARMACY.REC.1399.268). All participants signed written informed consent form after explaining the objectives of the study and ensuring the confidentiality of data for them.

## Results

In all 122 women with multiple scholars were entered onto the study. The mean age of women was 36.3 ± 7.40 years, and the majority (52%) had a university degree. The mean disease duration was 7.95 ± 6.22 years. Some characteristics of the participants are shown in Table 1.

**Table 1:** The characteristics of participants (*n* = 122)

	Mean (SD)	No. (%)
Age	36.3 (7.40)	
Husband’s age	40.7 (8.81)	
Education		
Primary		12 (10)
Secondary		46 (38)
Higher		64 (52)
Husband's education		
Primary		16 (13)
Secondary		51 (42)
Higher		55 (45)
Employment status		
Employed		79 (64.7)
Housewife		43 (35.3)
Husband’s employment status		
Employed		91 (74.6)
Unemployed		31 (25.4)
Economic status		
Good		23 (18.9)
Intermediate		76 (62.2)
Poor		23 (18.9)
Duration of marriage (years)	13.28 (9.86)	
Pregnancies	1.0 (1.0)	
Number of children	1.0 (0.82)	
Duration of the disease (years)	7.95 (6.22)	
Number of sexual intercourses before MS (per week)	1.93 (1.57)	
Number of sexual intercourses after MS (per week)	1.60 (1.20)	
Disease course		
Relapsing-remitting MS		101(82.8)
Secondary-progressive MS		8 (6.6)
Primary-progressive MS		13 (10.6)
Receiving sexual counseling		
Yes		14 (11.5)
No		108 (88.5)

The detailed descriptive statistics for sexual satisfaction and sexual intimacy scores are presented in Table 2. Overall the mean score of sexual satisfaction was 89.3 ± 18.6, and the mean score of sexual intimacy was 80.5 ± 17.2.

**Table 2:** Sexual satisfaction and sexual intimacy in participants (*n* = 122)

	Test range	Sample range	No. (%)	Mean (SD)
Sexual satisfaction				
Total score	25–125	37–125	122 (100)	89.3 (18.6)
High	90–125	90–125	50 (41)	108.6 (9.1)
Moderate	60–90	60–89	67 (54.9)	77.5 (6.4)
Poor	< 60	53–58	5 (4.1)	52.4 (8.8)
Sexual intimacy				
Total score	30–120	38–117	122 (100)	80.5 (17.2)
High	> 90	91–117	37 (30.3)	101.8 (7.9)
Moderate	60–90	61–88	72 (59.0)	74.4 (7.4)
Poor	< 60	38–59	13 (10.7)	53.3 (6.1)

The majority of women with MS (*n* = 71, 58.2%) used emotion-focused coping strategies in dealing with their disease, and the highest mean score was for scape-avoidance strategy (Mean = 13.29, SD = 5.40). The remaining 41.8% (*n* = 51) of the patients used problem-focused coping strategies, and the highest mean score was for positive reappraisal strategy (Mean = 10.50, SD = 4.96). The patients’ coping strategies scores are presented in Table 3.

**Table 3:** Coping strategies in participants (*n* = 122)

	Test range	Sample range	Mean (SD)
Emotion-focused coping strategies (*n* = 71)			
Total score	0–81	9–78	43.59 (14.67)
Confrontive coping	0–18	1–18	9.70 (3.95)
Distancing	0–18	1–18	9.71(4.05)
Escape-avoidance	0–24	1–24	13.29 (5.40)
Self-controlling	0–21	1–21	10.87 (4.80)
Problem-focused coping strategies (*n* = 51)			
Total score	0–69	2–69	35.34 (15.05)
Seeking social support	0–18	0–18	9.21 (4.57)
Accepting responsibility	0–12	0–12	6.35 (2.98)
Planful problem-solving	0–18	0–18	9.27 (4.34)
Positive reappraisal	0–21	0–21	10.50 (4.96)

As shown in Table 4, the results obtained from two independent samples *t*-test showed that sexual satisfaction was significantly higher in women with problem-focused coping strategy than women using emotion-focused coping strategy (*P* = 0.001). In terms of sexual intimacy, the difference between the two groups was not statistically significant (*P* = 0.570).

**Table 4:** Relationship between total score of sexual satisfaction and sexual intimacy with coping strategies in participants

	Problem- focused coping strategies (*n* = 51)	Emotion- focused coping strategies (*n* = 71)	*P*-value*
	Mean (SD)	Mean (SD)	
Sexual satisfaction	95.60 (19.2)	84.71 (16.99)	0.001
Sexual intimacy	81.60 (20.0)	76.74 (14.88)	0.570

*SD* standard deviation

^*^Derived from independent *T* test

Table 5 shows the results obtained from the multivariable logistic regression analysis indicating a significant negative association between likelihood of higher sexual satisfaction and marriage duration (OR = 0.891, 95% CI 0.808–983, *P* = 0.021), a significant positive association with frequency of sexual intercourse after MS (OR = 1.827, 95% CI 1.115–2.992, *P* = 0.017), and problem-focused coping strategy (OR = 1.114, 95% CI 1.049–1.182, *P* < 0.0001)). Similar analysis showed that there was a significant positive association between higher sexual intimacy and frequency of sexual intercourse after MS (OR = 1.755, 95% CI 1.090–2.828, *P* = 0.021), and a significant negative association with higher emotion-focused coping strategy score (OR = 0.919, 95% CI 0.872–0.968, *P* = 0.001). The results are shown in Table 6.

**Table 5:** The results obtained from logistic regression analysis for association between independent variables and likelihood of higher sexual satisfaction

	Odds ratio	95% CI	*P*
Age	1.068	0.954–1.194	0.254
Education			
Higher	1.0 (ref.)		
Primary/secondary	1.232	0.245–6.196	0.800
Marriage duration	0.891	0.808–0.983	0.021
Number of children	1.288	0.562–2.954	0.550
Frequency of sexual intercourse per week			
Before MS	0.877	0.609–1.262	0.480
After MS	1.827	1.115–2.992	0.017
Sexual problems			
No	1.0 (ref.)		
Yes	0.608	0.234–1.578	0.306
Problem-focused	1.114	1.049–1.182	< 0.0001
Emotion-focused	0.956	0.906–1.010	0.107

**Table 6:** The results obtained from logistic regression analysis for association between independent variables and likelihood of higher sexual intimacy

	Odds ratio	95% CI	*P*
Age	1.002	0.905–1.110	0.965
Education			
Higher	1.0 (ref.)		
Primary/secondary	0.501	0.079–3.164	0.462
Marriage duration	0.933	0.846–1.029	0.165
Number of children	1.239	0.585–2.623	0.575
Frequency of sexual intercourse per week			
Before MS	1.103	0.813–1.496	0.528
After MS	1.755	1.090–2.827	0.021
Sexual problems			
No	1.0 (ref.)		
Yes	0.995	0.410–2.417	0.992
Problem focused	1.045	0.994–1.099	0.085
Emotion focused	0.919	0.872–0.968	0.001

## Discussion

The findings from this study indicated that there was a significant positive relationship between problem-focused coping strategies and sexual satisfaction, while sexual intimacy did show a significant negative association with emotion-focused coping strategy.

We found that the majority of participants had moderate sexual satisfaction (54.9%). In a study by Zamani et al., sexual dissatisfaction due to sexual dysfunction in women with MS was relatively low [14]. This difference can be attributed to the fact that we used a specific measure of sexual satisfaction while they used the female sexual functioning index. One of the main complications of MS is sexual disorders such as reluctance to intercourse and impaired orgasm in many affected women [6, 14]. The disorders usually occur due to muscle weakness [33], urinary incontinence [24], fatigue, anxiety, and depression [1]. These all can decrease self-confidence and distortion of the appropriate feminine image of their sexual abilities compared to women without MS. With the mentality of inability to sexually satisfy the spouse, inability to play an effective role in sexual intercourse, and creating a mental-psychological barrier, these disorders prevent sexual intercourse and significantly reduce the sexual satisfaction [34].

The majority of participants had moderate sexual intimacy (59.0%). It seems that sexual disorders caused by MS could intensify a feeling of guilt [1], and such feeling might reduce a woman’s sense of security and create an anxious environment, and ultimately lead to not sharing the feelings, needs, and sexual perceptions with their sexual partner, which in turn leads to more separation between couples and diminishes sexual relations between them [31]. Indeed, living in a safe and stress-free environment helps to improve sexual relationships and intimacy [16, 33].

In the present study, less than half of the participants used problem-focused coping strategies, and the most frequent strategy was positive reappraisal which refers to the acceptance and positive interpretation of the disease. Coping by positive reappraisal is shown to be correlated positively with psychological well-being [35–37]. Implementing this strategy assists patients to redefine their condition more positively and adapt to stressful situations [38]. Some studies have shown that using this strategy brings out more flexibility confronting the disease or stressful conditions [39–41]. Patients who use problem-focused coping strategies in dealing with their disease achieved a relative acceptance of the existing conditions over time, considering all the limitations and complications of MS [23] and it has strengthened their use of problem-focused strategies. Interpreting stressful events is more important than the events themselves, which helps patients, respond to disease as a source of stress [33].

According to the results, more than half of the participants (58.2%) used emotion-focused coping strategies, especially the escape-avoidance strategy. Emotion-focused coping is always associated with weaker levels of compromise and higher levels of distress. In such situations, to change the source of stress and negative feelings and emotions caused by the disease, the patient tries to deny the disease or overcome it through daydreaming, conscious self-occupation, and participating in an alternative activity [16]. Acceptance of the disease, psychological complications, physical disabilities and mobility limitations are difficult for patients [42]. As such, the easiest and most accessible coping style is to resort to coping strategies based on expressing emotions, avoiding the source of the problem and accepting reality [22]. Hence, patients move away from rational thinking and conscious behavior [16]. Many previous studies have pointed to the regulating and modulating roles of coping strategies on the level of anxiety, stress and depression in patients, and it has been found that if these strategies are based on awareness and cognition, the patient will have higher peace of mind, efficiency and overall mental health [33].

The study results showed that sexual satisfaction was higher in women with problem-focused strategies. Perhaps applying a problem-focused coping strategy might lead patients to deal with issues more rationally and respond better to stressors or the disease [23, 24]. Therefore, she can accept the disease easier and adapt to the situation and does not have excessive expectations of herself, and ultimately with better mental health, she will have higher health with better sexual health [22]. In such a situation, the person will be at a healthier level of social relationships and consequently in a better situation of marital and sexual relationships because mental health and sexual health are strongly related to each other [33]. Marital and sexual relationships are considered as protective factors against psychological and physiological problems [14]. A woman using strategies to deal with her disease will have a lower stress level, be more successful in establishing relationships, and experience a higher sense of satisfaction, peace of mind and mental health. This manifests itself in all aspects of her personal and social life and its most important consequences are a successful sexual relationship, a good level of sexual health, and sexual satisfaction [33]. Therefore, as an individual strengthens the use of problem-focused coping strategies, it indirectly helps to increase sexual satisfaction [22, 23].

The findings indicated a significant negative relationship between emotion-focused coping strategy and sexual intimacy. In addition, marital relationships, regardless of sexual interactions, can affect sexual intimacy because sexual intimacy is dynamic and can change over time [43]. It is argued that a better understanding of sexual intimacy might need to incorporate interpersonal dynamics into current models of sexual activities between partners [44, 45]. In fact, it can be said that attachment and empathy between couples and deep emotional connections play a greater role in creating sexual intimacy than the type of coping strategy. Other studies on Iranian women with MS showed the role of demographic factors (education and employment status) and implementing support strategies by the husband for improving sexual function [27, 46]. The desire for intimacy has biological roots and for most people lasts from birth to death. Intimacy in marital relationships has strong emotional and social aspects and is based on acceptance, satisfaction and love [16]. For instance, physical intimacy or emotional intimacy that could reflect the ‘innermost’ feelings are necessary components of a healthy relationship and evidently higher levels of intimacy are associated with more sexual desire [44]. Other studies have also emphasized the relationship between sexual intimacy, and quality of marital life [42, 47].

## Strengths and limitations

One of the strengths of the present study was the investigation of the relationship between important factors of sexual health, namely sexual satisfaction and intimacy with coping strategies in women with MS, which rarely was considered in previous studies. Among the study limitations is the fact that we recruited the study samples from one setting, and there was no comparison group. Thus the generalization of the results should be avoided. In addition, since we only included sexually active patients, a selection bias might be occurred, as the study does not include women who ceased having sex due to MS. Finally, since we did not collect the data for those who might be used both coping strategies; the results should be interpreted with caution. Perhaps future investigations should assess the outcome of different interventions that are seeking to improve different coping strategies among this population.

## Conclusion

The results suggest that using problem-focused coping strategy in women with MS significantly increases sexual satisfaction, and using emotion-focused strategy significantly decreases sexual intimacy. Indeed, to promote sexual satisfaction and intimacy in women with multiple sclerosis health providers might focus on improving women’s skills on using problem-focused coping strategy.

## Data Availability

The data sets used and/or analyzed during the current study available from the corresponding author on reasonable request.

## Abbreviation

MS: Multiple sclerosis
